# Association between domain-specific physical activity and diabetes in Korean adults

**DOI:** 10.1038/s41598-021-92560-x

**Published:** 2021-06-22

**Authors:** Eun-Byeol Lee, Sunghyun Hong, Jihee Min, Dong-Hyuk Park, Wonhee Cho, Sang-Hoon Suh, Hae-Dong Lee, Han-Joo Lee, Heejin Kimm, Sun Ha Jee, Eun Seok Kang, Dong Hoon Lee, Justin Y. Jeon

**Affiliations:** 1 Department of Sport Industry Studies, Yonsei University, 50 Yonseiro, Seodaemun-gu, Seoul, 03722 South Korea; 2 Exercise Medicine Center for Diabetes and Cancer Patients, ICONS, Yonsei University, Seoul, South Korea; 3 Frontier Research Institute of Convergence Sports Science, Yonsei University, Seoul, South Korea; 4 Department of Physical Education, Yonsei University, Seoul, South Korea; 5 Graduate School of Public Health, Institute for Health Promotion, Yonsei University, Seoul, South Korea; 6 Department of Internal Medicine, Yonsei University College of Medicine, Seoul, South Korea; 7 Department of Nutrition, Harvard T.H. Chan School of Public Health, Boston, MA 02115 USA

**Keywords:** Diabetes, Epidemiology, Obesity

## Abstract

This study aimed to investigate the association between domain-specific physical activity (PA) and diabetes in Korean adults. We analyzed 26,653 men and women (aged > 18 years) from the Korea National Health and Nutrition Examination Survey (2014–2018). PA was measured using a validated Global PA Questionnaire. Multivariable logistic regression was used to estimate odds ratios (ORs) and 95% confidence intervals (CIs) after adjustment for various confounders. Transport PA accounted for the majority of total PA (46%, men; 58%, women), followed by leisure-time PA (30%; 22%) and work PA (24%; 20%). In men, ORs (95% CI) of diabetes comparing ≥ 600 metabolic task of equivalent (MET)-min/week vs. no activity were 0.82 (0.71–0.95) for leisure-time PA, 0.85 (0.75–0.96) for transport PA, and 0.88 (0.78–0.99) for leisure-time + transport PA. In women, ORs (95% CI) of diabetes comparing the same groups were 0.73 (0.60–0.89) for leisure-time PA, 0.97 (0.85–1.10) for transport PA, and 0.88 (0.78–1.00) for leisure-time + transport PA. However, work PA showed no association with diabetes. In conclusion, leisure-time PA was inversely associated with diabetes in both men and women, while transport PA was inversely associated only in men. But work PA was not associated with diabetes in Korean adults.

## Introduction

The rising burden of type 2 diabetes is one of the major concerns in healthcare^1^. In 2017, approximately 462 million individuals (6,059 per 100,000) were affected by type 2 diabetes, which is 6.3% of the world’s population and expected to increase up to 7,079 individuals per 100,000 by 2030^2^. Asia is a major area of the rapidly emerging type 2 diabetes global epidemic, with China and India the top two epicenters^1,3^. Although genetic predisposition is an important contributor for increased susceptibility to type 2 diabetes, an unhealthy diet and a sedentary lifestyle are two important drivers of the current global epidemic^4–6^.

Increasing physical activity (PA) along with other lifestyle modification is one of the most effective ways to prevent type 2 diabetes as well as glycemic control for those who already have diabetes. Studies consistently showed that leisure-time PA, vigorous PA and resistance exercise were associated with reduced risk of type 2 diabetes^7–10^. More specifically, lifestyle intervention studies including aerobic and resistance training were shown to be effective in managing weight and improving fasting glucose^7,11^. A twin study from Finland which followed 146 twin pairs for 30 years showed that active twins were at lower risk of developing type 2 diabetes compared to inactive co-twins^12^. Several studies also suggested that intensity and type of PA were associated with diabetes prevention. For example, a significant decrease in fasting glucose and risk of diabetes was observed among overweight or obese participants who performed high-intensity PA^13^. Davy et al. reported that overweight/obese pre-diabetic individuals improved their abnormal blood glucose response after a 15-month resistance exercise intervention^14^. Furthermore, a large prospective cohort study showed that participation in resistance exercises is associated with reduced risk of developing diabetes by 34% over 18 years^15^. However, little is known whether non-leisure PA such as work PA and transport PA would affect the risk of type 2 diabetes^16–18^.

In this context, global PA questionnaire (GPAQ) has unique merit since it covers different domains of PA including PA at work, travel to and from places (transport), and leisure-time activities^19^. The use of the GPAQ not only survey different domains of PA, but also different intensity of PA. In South Korea, GPAQ was translated into Korean languages and Korea Centers for Disease Control and Prevention (KCDC) in their National Health and Nutrition Examination Survey (KNHANES) implanted a few other important questions such as the number of days of resistance exercise participation^20^. This provides unique opportunity to examine whether PA at different domains with different intensity in combination with resistance exercise participation would be associated with diabetes.

Therefore, the purpose of the current study was to examine the association of domain and intensity specific PA and participation of resistance exercise with diabetes among 26,653 Korean adults.

## Materials and methods

### Study participants

We used a large representative data from the KNHANES 2014–2018^21^. The target population of KNHANES consists of Korean citizens living in Korea. The sampling plan follows a multistage clustered probability design. KNHANES survey started in 1998, conducted every three years. Then, KNHANES was conducted every year starting from 2007. Since GPAQ was implemented to assess domain specific PA starting in 2014, we used data from the KNHANES 2014–2018. Among 31,310 participants included in 2014–2018 surveys, a total of 26,653 participants were used in the final analysis after excluding those younger than 18 years, with no data on PA and diabetes or whose fasting time was less than 7 h. All participants provided informed consent and this study was approved by the Research Ethics Review Committee of the Korea Centers for Disease Control and Prevention.

### Data collection

The KNHANES data was collected by using self-reported questionnaires or research staff interviews. The detailed procedures of selection of households and methods of interviews were described previously^7^. Once appropriate households in the survey area are verified, the KCDC distributed a notice of the selection of household certification one month prior to the investigation. One week before the investigation, precautions and locations were introduced by phone to set up an appointment. The subjects were weighted according to the region so that they could represent the entire population of Korea, and residents and foreigners of collective facilities such as nursing homes, military, and prisons were excluded for the representation of the sample.

Demographic, socioeconomic, and lifestyle data were collected using self-reported questionnaires. Anthropometry (height, weight and waist circumference), metabolic risk factors (blood pressure, fasting glucose, HbA1c and lipid profile) were measured or obtained from blood lab tests. Family history of diabetes was asked if any of the parents or siblings had a history of diabetes. Smoking status was asked if participants are a current, past or never smoker. Frequency of drinking during the past year was assessed, and the frequency was categorized into less or more than once a month for the last year. Income was categorized into quartiles. The highest academic degree including elementary school, middle school, high school, or college graduate or higher was surveyed.

### Physical activity

In KNHANES, PA was measured through the GPAQ^19^, which was translated into Korean and its validity and reliability were verified^20^. The survey questions consist of a total of 16 questions, which collect information on PA participation in three different domains as well as sedentary behavior. These domains are work PA, transport PA, and leisure-time PA. Work PA and leisure-time PA consist of a total of 12 questions: six questions for each domain. Three questions are on transport PA and one question is on sedentary behavior. Total PA was calculated by summing all domain-specific PA.

For descriptive analysis, we presented domain-specific PA in minutes per week. For main analysis, we used MET-minutes per week after assigning MET values (4 METs for moderate-intensity and 8 METs for vigorous-intensity) to incorporate the intensity of PA. In addition to GPAQ, participation of resistance exercise, assessed as the number of days participating in resistance exercise per week was surveyed.

### Diabetes

Participants with diabetes in our study were defined by being diagnosed by a medical doctor, currently taking medications or insulin for the treatment of diabetes, or having limited activity due to diabetes. This information was collected from computer assisted personal interviewing (CAPI) method. Furthermore, participants were classified as diabetes when their fasting blood glucose was ≥ 126 mg/dL or HbA1c ≥ 6.5% (48 mmol/mol)^22^. Blood collection was conducted after fasting for at least eight hours at the mobile examination center through a screening investigation and was conducted and analyzed by experts consisting of nurses and clinical pathologists^21^.

We identified 3,661 participants who met our diabetes diagnosis criteria and the blood index criteria. Of these, 1,096 were undiagnosed diabetes, which had not received a doctor’s diagnosis but could be determined through blood indicators.

### Statistical analysis

Descriptive analyses were used to present the characteristics of participants. To compare the differences in characteristics, we conducted independent *t*-test for continuous variables and Chi-squared (*χ*^2^-test) for categorical variables. Multivariable-adjusted logistic regression was used to estimate the odds ratios (ORs) and 95% confidence intervals (CIs) of the association between domain-specific PA and diabetes. To adjust for potential confounders, we included predefined covariates including age, family history of diabetes, alcohol consumption, smoking status, income, education, menopausal status (for women only) and sedentary time. We ran an additional model further adjusting for body mass index (BMI), a potential mediator of the relationship between PA and diabetes. Total and each domain-specific PAs (leisure, work, transport, leisure + transport) were categorized into three groups (0, < 600, ≥ 600 METs-minutes/week) based on the current PA recommendation guidelines. The 600 METs-minutes/week shown here followed the ACSM guidelines recommending adults participate in moderate-intensity PA at least 150 min or vigorous-intensity PA at least 75 min per week. Having a large number of inactive participants, we also modeled domain-specific PA as a binary variable (yes or no) to increase power.

For secondary analyses, we further examined the association of total and each domain-specific PAs by intensity (moderate vs. vigorous) with diabetes. Because detailed PA intensity information was available only for work and leisure-time PA (not transport PA), vigorous-intensity PA included work and leisure-time PAs, while moderate-intensity PA included work, leisure, and transport PAs. Vigorous and moderate domain-specific PAs were used as a binary variable (yes or no) due to limited number of participants with high levels of vigorous or moderate PA. Moreover, we tested whether engaging in resistance training (yes or no; 1-day increase) was associated with diabetes. Lastly, we conducted a subgroup analysis to explore whether the associations of domain-specific PA and diabetes differ by sociodemographic and lifestyle risk factors. To assess the robustness of our findings, we also conducted a sensitivity analysis restricting to undiagnosed diabetes cases. All analyses were performed separately by sex and all statistical analyses were done using SPSS 25 version (IBM Co&Kr., Chicago, US).

### Ethics approval

This research study was conducted retrospectively from data obtained for clinical purposes. The questionnaire in this study was approved by the Research Ethics Review Committee of the Korea Centers for Disease Control and Prevention (IRB). This committee is operated on the basis of the Helsinki Declaration, as well as the Standards Operation Guide for the Research Ethics Review Committee of the Centers for Disease Control and Prevention.

## Results

**Figure 1:**
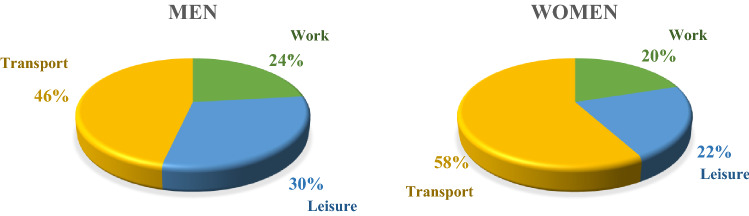
Percentage by domain of physical activity.

Table 1 presents the characteristics of participants by sex. The mean of age and body mass index were 51 years and 24 kg/m^2^ for both men and women. The average total PA level was 262 min/week for men and 198 min/week for women. When we examined the percentage of domain-specific PA, transport PA accounted for the majority of total PA (46% for men and 58% for women), followed by leisure-time PA (30% for men and 22% for women) and work PA (24% for men and 20% for women) (Fig. 1).

**Table 1:** Characteristics of participants.

	Total	Men	Women
	n = 26,653	n = 11,478	n = 15,175
Age (years)	51.2 ± 16.7	51.1 ± 16.9	51.29 ± 16.7
BMI (kg/m^2^)	23.9 ± 3.5	24.4 ± 3.3	23.51 ± 3.6
**DM, N (%)**			
Diagnosed	2565 (9.6)	1250 (10.9)	1315 (8.7)
Undiagnosed		589 (5.8)	507 (3.7)
Family history, N (%)	5948 (22.3)	2331 (20.3)	3617 (23.8)
Missing	785 (2.9)	387 (3.4)	398 (2.6)
Menopause, N (%)			7813 (51.5)
**Smoking, N (%)**			
Current	4750 (17.8)	4022 (35.0)	728 (4.8)
Past	5594 (21.0)	4729 (41.2)	865 (5.7)
Never	16,133 (60.5)	2658 (23.2)	13,475 (88.8)
Missing	176 (0.7)	69 (0.6)	107 (0.7)
**Alcohol consumption, N (%)**			
< once/1 month	12,231 (45.9)	3306 (28.8)	8925 (58.8)
≥ once/1 month	14,264 (53.5)	8109 (70.6)	6155 (40.6)
Missing	158 (0.6)	63 (0.6)	95 (0.6)
**Income, N (%)**			
Q1	4997 (18.7)	1939 (16.9)	3058 (20.2)
Q2	6483 (24.3)	2745 (23.9)	3738 (24.6)
Q3	7412 (27.8)	3277 (28.6)	4135 (27.2)
Q4	7684 (28.8)	3491 (30.4)	4193 (27.6)
Missing	77 (0.4)	26 (0.2)	51 (0.4)
**Education, N (%)**			
Elementary school	4985 (18.7)	1405 (12.2)	3580 (23.6)
Middle school	2914 (10.9)	1221 (10.6)	1693 (11.2)
High school	7202 (27)	3170 (27.6)	4032 (26.6)
Over College	11,479 (43.1)	5650 (49.3)	5829 (38.4)
Missing	73 (0.3)	32 (0.3)	41 (0.2)
**Physical activity (min/wk)**			
Vigorous PA	25.9 ± 128.6	42.5 ± 175	13.3 ± 74.3
Moderate PA	199.9 ± 334.4	219.6 ± 352.9	185.1 ± 318.9
**At work (min/wk)**			
Vigorous PA	8.1 ± 102.2	14.7 ± 142.8	3.1 ± 53.5
Moderate PA	40.1 ± 229.4	46.2 ± 229.6	35.5 ± 229.2
**Leisure-time (min/wk)**			
Vigorous PA	17.8 ± 73.3	27.9 ± 94.8	10.2 ± 49.9
Moderate PA	41.8 ± 106.4	52.1 ± 121.1	34.0 ± 93.1
Transport PA (min/wk)	118.4 ± 198.2	121.5 ± 220.1	116.0 ± 179.7
Sedentary time (min/wk)	475.2 ± 214.8	479.3 ± 216.9	472.0 ± 213.1

Data are presented as mean ± standard deviation or number (percentage). Abbreviation: DM, Diabetes mellitus; PA, physical activity; BMI, body mass index; WC, waist circumference; FG, fasting glucose; TG, triglyceride; SBP, systolic blood pressure; DBP, diastolic blood pressure; TC, total cholesterol; HDL-C, high-density lipoprotein cholesterol; LDL-C, low-density lipoprotein cholesterol; DM, Diabetes mellitus; PA, physical activity; Q, quintile.

Leisure-time PA was significantly inversely associated with odds of diabetes in both men and women, while transport PA was only inversely associated in men (Table 2). In multivariable-adjusted models (Model 2), ORs (95% CIs) of diabetes comparing ≥ 600 MET-min/week vs. no activity were 0.82 (0.71–0.95) for leisure-time PA, 0.85 (0.75–0.96) for transport PA, and 0.88 (0.78–0.99) for leisure-time + transport PA in men. In women, ORs (95% CIs) of diabetes comparing the same groups were 0.73 (0.60–0.89) for leisure-time PA, 0.97 (0.85–1.10) for transport PA, and 0.88 (0.78–1.00) for leisure-time + transport PA. However, work PA showed no association while total PA including all three domains of PA showed a marginally non-significant association with odds of diabetes. Additional adjustment for BMI did not materially changed the results (Model 3). In sensitivity analyses, using the binary domain-specific PA (yes or no) (Supplementary Table 1) or restricting the analysis to undiagnosed diabetes showed consistent results (Supplementary Tables 2–3). In subgroup analyses, we found no evidence that the association of domain-specific PA with diabetes differ by age, BMI, family history of disease, income, education, smoking, alcohol intake, and sitting time (Supplementary Tables 4–7).

**Table 2:** Odds ratio (95% CI) of diabetes according to domain-specific physical activity.

	Men		Model 1	Model 2	Model 3	Women		Model 1	Model 2	Model 3
	N^a^	DM^b^	OR (95% CI)	OR (95% CI)	OR (95% CI)	N^a^	DM^b^	OR (95% CI)	OR (95% CI)	OR (95% CI)
**Total PA (METs-min/wk)**										
0	3523	670	ref					4776	746	ref
< 600	2302	402	0.99 (0.85–1.14)	0.99 (0.85–1.14)	1.00 (0.86–1.15)	3913	511	0.98 (0.86–1.11)	0.99 (0.87–1.13)	1.02 (0.89–1.16)
≥ 600	5653	767	0.90 (0.80–1.02)	0.90 (0.80–1.02)	0.90 (0.80–1.02)	6486	565	**0.83** (0.73–0.94)	**0.85** (0.75–0.96)	0.90 (0.79–1.02)
**WPA (METs-min/wk)**										
0	10,218	1683	ref					13,930	1721	ref
< 600	374	54	1.15 (0.84–1.56)	1.15 (0.84–1.60)	1.16 (0.85–1.58)	473	42	0.92 (0.65–1.28)	0.93 (0.66–1.30)	0.94 (0.66–1.32)
≥ 600	885	102	1.05 (0.84–1.31)	1.06 (0.84–1.32)	1.02 (0.81–1.28)	762	59	0.94 (0.70–1.26)	0.95 (0.70–1.27)	0.88 (0.65–1.19)
**LPA (METs-min/wk)**										
0	7558	1368	ref					11,698	1597	ref
< 600	1369	188	1.00 (0.84–1.19)	1.00 (0.84–1.19)	0.96 (0.81–1.15)	1474	98	**0.69** (0.56–0.86)	**0.70** (0.56–0.88)	**0.73** (0.58–0.91)
≥ 600	2545	283	**0.82** (0.71–0.94)	**0.82** (0.71–0.95)	**0.80** (0.69–0.93)	1999	127	**0.72** (0.59–0.87)	**0.73** (0.60–0.89)	**0.79** (0.65–0.97)
**TPA (METs-min/wk)**										
0	5477	938	ref					6233	835	ref
< 600	2552	436	0.98 (0.86–1.12)	0.98 (0.86–1.12)	1.01 (0.88–1.15)	4352	536	1.03 (0.91–1.16)	1.04 (0.92–1.18)	1.07 (0.94–1.21)
≥ 600	3427	465	**0.85** (0.75–0.96)	**0.85** (0.75–0.96)	**0.86** (0.75–0.97)	4545	451	0.94 (0.83–1.07)	0.97 (0.85–1.10)	1.00 (0.87–1.14)
**LPA + TPA (METs-min/wk)**										
0	3859	720	ref					5069	767	ref
< 600	2378	414	0.99 (0.96–1.13)	0.99 (0.86–1.13)	0.99 (0.86–1.14)	4035	513	0.99 (0.87–1.13)	1.01 (0.88–1.14)	1.03 (0.91–1.18)
≥ 600	5238	705	**0.87** (0.78–0.99)	**0.88** (0.78–0.99)	**0.87** (0.77–0.99)	6068	541	**0.86** (0.76–0.97)	**0.88** (0.78–1.00)	0.94 (0.82–1.07)

Model 1: Adjusted for age, Model 2: Adjusted for Model1 + DM family history, alcohol consumption, smoking, income, education, menopause, sedentary time, Model 3: Adjusted for Model 2 + BMI, Bold is p < 0.05, Abbreviation: DM, diabetes mellitus; PA, physical activity; WPA, work physical activity; LPA, leisure-time physical activity; TPA, transport physical activity; ref, reference.

^a^ Number of participants in each category.

^b^ Number of diabetes cases in each category.

We further investigated whether the domain-specific PA by intensity was associated with odds of diabetes (Table 3). We observed that either moderate or vigorous work PA was not associated odds of diabetes. However, vigorous leisure-time PA was more strongly inversely associated with odds of diabetes in men (OR: 0.68, 95% CI: 0.56–0.82), while moderate leisure-time PA was more strongly inversely associated with odds of diabetes in women (OR: 0.76, 95% CI: 0.64–0.89). When we examined the association between resistance exercise and odds of diabetes, we found that participants with any resistance exercise had lower odds of diabetes in men (OR: 0.80, 95% CI: 0.71–0.90) and women (OR: 0.80, 95% CI: 0.68–0.95) (Table 3). Moreover, increase in one-day of resistance exercise per week was associated with lower odds of diabetes in men (OR: 0.96, 95% CI: 0.93–0.99) and women (OR: 0.96, 95% CI: 0.91–1.00) after adjusting for total PA (Supplementary Tables 8).

**Table 3:** Odds ratio (95% CI) according to domain-specific physical activity by intensity and type.

	Men		Model 1	Model 2	Model 3	Women		Model 1	Model 2	Model 3
	N^a^	DM^b^	OR (95% CI)	OR (95% CI)	OR (95% CI)	N^a^	DM^b^	OR (95% CI)	OR (95% CI)	OR (95% CI)
**Total VPA (min/wk)**										
No	9404	1660	ref			13,831	1765	ref		
Yes	2074	179	**0.76** (0.64–0.90)	**0.79** (0.66–0.94)	**0.74** (0.62–0.88)	1157	57	**0.70** (0.53–0.92)	0.81 (0.61–1.08)	0.77 (0.58–1.02)
**Work VPA (min/wk)**										
No	11,120	1794	ref			15,012	1802	ref		
Yes	354	45	1.26 (0.90–1.76)	1.22 (0.87–1.72)	1.29 (0.91–1.82)	148	13	0.94 (0.52–1.72)	0.82 (0.44–1.51)	0.86 (0.46–1.60)
**Leisure-time VPA (min/wk)**										
No	9635	1694	ref			14,126	1774	ref		
Yes	1837	144	**0.69** (0.57–0.83)	**0.68** (0.56–0.81)	**0.68** (0.56–0.82)	1043	45	**0.64** (0.47–0.88)	0.74 (0.54–1.01)	0.75 (0.55–1.03)
**Total MPA (min/wk)**										
No	3783	695	ref			4913	758	ref		
Yes	7695	1144	0.95 (0.85–1.06)	0.95 (0.85–1.06)	0.95 (0.85–1.06)	10,262	1064	**0.90** (0.80–1.00)	0.95 (0.85–1.06)	0.96 (0.86–1.07)
**Work MPA (min/wk)**										
No	10,312	1694	ref			13,972	1726	ref		
Yes	1162	144	1.08 (0.89–1.31)	1.05 (0.86–1.28)	1.05 (0.86–1.28)	1188	91	0.93 (0.74–1.17)	0.91 (0.72–1.15)	0.93 (0.73–1.17)
**Leisure-time MPA (min/wk)**										
No	8170	1412	ref			12,029	1618	ref		
Yes	3298	425	0.95 (0.84–1.07)	0.93 (0.82–1.05)	0.93 (0.82–1.05)	3140	201	**0.70** (0.59–0.82)	**0.75** (0.64–0.89)	**0.76** (0.64–0.89)
**Transport PA (min/wk)**										
No	5477	649	ref			6233	619	ref		
Yes	5979	600	0.91(0.82–1.01)	0.91(0.82–1.01)	0.92(0.83–1.03)	8897	694	0.99(0.89–1.10)	1(0.90–1.12)	1.03(0.92–1.15)
**Resistance exercise**										
No	7739	1353	ref			12,705	1637	ref		
Yes	3719	481	**0.79** (0.71–0.89)	**0.79** (0.70–0.89)	**0.80** (0.71–0.90)	2452	180	**0.73** (0.61–0.86)	**0.79** (0.67–0.94)	**0.80** (0.68–0.95)

Model 1: Adjusted for age, Model 2: Adjusted for Model1 + DM family history, alcohol consumption, smoking, income, education, menopause, sedentary time, BMI, Model 3: Adjusted for Model 2 + moderate or vigorous PA time, Bold is p < 0.05, Abbreviation: DM, diabetes mellitus; VPA, vigorous physical activity; MPA, moderate physical activity; ref, reference.

^a^Number of participants in each category.

^b^Number of diabetes cases in each category.

## Discussion

In this study, we examine the relationship between domain-specific PA and prevalence of diabetes among 26,653 Korean adults. We found no association between work PA and diabetes, whereas a significant inverse association was observed for higher levels of leisure-time PA, transport PA as well as resistance exercise. Higher transport PA was associated with lower prevalence of diabetes in men but no association was observed in women. Our study shows that most of the PAs performed by Koreans consist of transport PA, and that accounts for a large portion of the total PA. However, unlike other PA domains, the intensity of transport PA was not collected and all of transport PA was considered as moderate intensity. The observed sex differences in transport PA may be due to differences in intensity of transport PA between men and women. Compared to men, it is possible that the intensity of transport PA is not sufficiently achieved to be associated with lower risk of diabetes in women. However, when leisure-time and transport PA were considered together, weekly PA more than 600 METs-min were associated with reduced odds of diabetes in both men and women.

Interestingly, we observed lower prevalence of diabetes among participants engaging in any vigorous PA. In men, a significant association between vigorous leisure-time PA and prevalence of diabetes remained significant even after adjusting for BMI and moderate leisure-time PA. Gender-specific association between intensity and domain-specific PA was further observed, suggesting that diabetes was more strongly associated with vigorous leisure-time PA in men and moderate leisure-time PA in women. We further studied whether participation in any resistance exercise would be associated with diabetes and found significantly reduced prevalence of diabetes in both men and women.

Leisure-time and transport PA is associated with a decreased risk of diabetes^16,23^. However, the existing evidence for the association between work PA and diabetes risk is limited and inconsistent. One study showed that active participants with higher levels of work or transport PA had lower incidence of metabolic syndrome compared to those with a minimum level of PA^18^. In that study, the prevalence of diabetes tended to slightly increase as the amount of work PA increases in men, although the association was not statistically significant. The differences in these findings may be due to differences in definition and/or amount of domain-specific PA across different races/ethnicities or due to different physical conditions.

Previous studies have reported somewhat conflicting results; although there is a study that observed an association between work PA and reduced risk of diabetes^16,23^, most studies observe no association between work PA and risk of diabetes^24–26^. In our study, we observed no association between work PA and prevalence of diabetes in both men and women. Although most studies used the term ‘occupational PA’ and ‘work PA’ interchangeably, there is a difference between these two. When GPAQ refers to work PA, it is not limited to occupational PA. Work PA in GPAQ is defined as “the things that you have to do such as paid and unpaid work, household chores, harvesting food/crops, fishing or hunting for food, seeking employment”^27^. In our study, about 20–24% of our participants reported that there is any work PA, and only 11% of women who had diabetes reported that they have any work PA, probably due to older age in diabetic women. According to a 2017 study using the KNHANES, occupations of the participant were classified as manager/profession (17.1%), office job (12.9%), inoccupation (housewife/students) (35.1%), service industry (12%), engineer (11.8%), agriculture/forestry/fish (3.2%), and labor (7.9%). Moreover, 73% of employees worked in a job for pay. In this study, aerobic PA was represented as the sum of leisure-time and work PA, and both aerobic and muscular groups were represented. The groups were divided into aerobic physical activity, resistance exercise, aerobic and resistance combined and neither. Regardless of the occupations, the percentage of group not participating in any physical activity was the highest (over 50%), and participation in resistance exercise than aerobic and combined exercise was higher. In the case of office job, which is a typical sedentary group, more people participated in the resistance exercise (17.3%) than those who participated in the aerobic exercise (10.9%)^28^. It is still unclear as to why work PA is not associated with risk of diabetes in most studies including ours. However, lack of the association between work PA and health outcomes is not limited to diabetes but also observed in other chronic diseases such as metabolic syndrome, hypertension, and even cancer^25,29,30^.

Unlike work PA, leisure-time PA was associated with reduced prevalence of diabetes in the current study, in agreement with many previous studies^16,31–33^. It is well known that exercise reduces body fat mass and systemic inflammation as well as improves insulin sensitivity and glycemic control^34–37^ thus it is not surprising that people who participate in more leisure-time PA are less likely to develop diabetes. However, the inverse association between leisure-time PA and risk of diabetes is unlikely to be entirely explained by physiological pathways. Because most leisure-time PA is composed of sports and some kind of fun activities which have stress relieving components, psychological effects of leisure-time PA also exists^38^.

Interestingly, we further noticed in our study that, in addition to the amount of PA, the intensity of PA was associated with prevalence of diabetes with some gender differences. Vigorous leisure-time PA was associated with reduced odds of diabetes in men while moderate leisure-time PA was associated with reduced odds of diabetes in women even after adjusting for BMI and moderate/vigorous PA. In women, the relationship between vigorous leisure-time PA and diabetes was not statistically significant, but it showed a tendency to lower. Recently, Strain et al. studied 96,476 participants’ PA levels measured by accelerometer in relation to their mortality risk and reported that higher intensity PA had additional benefit, even when PA energy expenditure was identical^39^. Results from the current study along with previously reported study^40^ suggest that higher intensity PA should be recommended if people do not have any health problems which may be exacerbated during high-intensity PA.

Khaing Nang et al. reported that work and household-related activities contributed to overall PA than leisure-time or transport PA among Asians^41^. In our study, the proportion of transport PA was 46% and 58% in men and women, respectively. Interestingly, the proportion of transport PA increased up to 71% among women with diabetes. According to a survey of PA patterns for people aged 50 or older using data from the 2014 KNHANES, work PA, transport PA and leisure PA accounted for 13.6%, 80.9%, and 36.9%, respectively^42^. Koreans over the age of 50 had the highest PA related to transport PA. When the relation between transport PA and the risk of diabetes was examined in our study, we observed inverse association in men but not in women. However, when leisure-time and transport PA were added up, there was a significant inverse association between leisure-time + transport PA and the odds of diabetes. It is unclear as to why there is a gender difference between the amount of transport PA and the odds of diabetes, but it is noteworthy that the proportion of transport PA in total PA is much greater in women. Combining results from our study and others, transport PA could be used as an effective strategy to increase PA and consequently to prevent diabetes^43^.

The fact that Korea has one of the longest average commute time among OECD countries may explain why transport PA accounts for the largest part of total PA in Koreans. It has increased by six minutes over the past 20 years from 29.6 min in 1995 to 35.4 min in 2015. In addition, the number of people using public transportation (e.g., bus, subway or train) increased to 43.2% in 2017, compared to 38.8% in 2007. Internationally, the percentage of public transportation use in Korea is very high, which is more than twice as high as that of OECD countries^44^. In the GPAQ, PA of moving places (transport PA) asks about experience of walking for more than 10 min, riding a bicycle, or using public transportation during the day. On average, Koreans take longer commuting hours than other countries, thus transport PA is likely measured higher. Also, according to a 2015 survey conducted by the Ministry of Land, Infrastructure and Transport, Korean men and women had different transport patterns which may provide some evidence for the observed gender difference in the association between transport PA and diabetes. For example, men had the highest percentage of personal automobile use (52.2%) followed by walking (24.2%) and public transportation (15.3%), while women had the highest percentage of walking (51.5%) followed by public transportation (27.7%) and personal automobile usage (18.1%)^45^.

Transport PA depends on the country or race. According to a study conducted to examine the level and trend of PA among adults in 122 countries, 31.1% were physically inactive and high-income countries had lower PA levels than low-middle income countries. Since the 2000s, a small number of people have walked to work in Switzerland, USA, and Australia (less than 4%). However, more than 20 percent people of China, Germany, and Sweden walked to work. China had the largest proportion of active commuting by walking or cycling (46.1%) and more than 30% for France, Germany, Netherlands and Sweden and more than 10% for Brazil, Finland, UK and Ireland and less than 5% for Australia, Switzerland and US. The percentage of PA in transportation to work reflects how safe it is to walk or ride a cycle in each country. The weather of each country can also have a big impact^46^. Colombia, Brazil, England, and Australia are increasing in recreational, leisure or sports-related PAs, and the participation rate of men is higher than women, similar to that in Korean^47–50^. The participation rate of Taiwanese adults in PA was similar to that of Korea and the highest leisure-time PA was shown among middle-aged people aged over 45 years^51^. In the study of Min et al., in most age groups in Korea, transport PA was higher than other domains of PA, regardless of their overall PA levels, while in men, leisure-time PA was higher in the 40–69 age group with high total PA. However, women had higher leisure-time PA (1,539.5 MET-min/wk) for the 40–59 age group with high total PA, but it was not much different from transport PA (1,520.6 MET-min/wk)^52^.

Accumulating evidence suggests that resistance exercise plays an important role in blood glucose management for diabetics^53–55^. Holten et al. reported that a 6-week resistance training significantly increased glucose clearance and improved key proteins in insulin signaling cascade including glucose transporter type (GLUT)-4 and insulin receptor in patients with type 2 diabetes^56^. Moreover, a 2-year randomized controlled trial of 137 people with pre-diabetes showed 65% and 74% reduced incidence of type 2 diabetes in the resistance training group and combined aerobic + resistance training group, respectively, compared to the control group^57^. A follow-up study of 10,680 older women found that higher frequency of aerobic and resistance training was associated with lower risk of developing type 2 diabetes^58^. In the present study, we demonstrated consistent results that engaging in resistance training was associated with 20% lower odds of diabetes, independent of total PA. Moreover, increasing one day of resistance training per week was significantly associated with 4–5% lower odds of diabetes in men and women. Our findings provide confirmatory evidence that participation in resistance training has additional benefits in the prevention of diabetes development.

## Strengths and limitations

Our study has several limitations. First, the KNHANES did not classify types of diabetes and thus we included both types 1 and 2 diabetes in the analyses. However, the prevalence of type 1 diabetes in Korea is very low (approximately 0.02%)^59^ thus it is unlikely that our findings would be affected by the small number of unidentified type 1 diabetes. Second, PA was measured using a self-reported questionnaire rather than an objective measure such as an accelerometer. Thus, measurement error in self-reported PA questionnaire, particularly non-leisure PAs like work and transport PAs, may have biased our results to the null. However, our PA questionnaire (GPAQ) was previously validated and has a unique advantage that allows assessing domain-specific PA which is the primary aim of our study. Also, the GPAQ has been widely used over 50 countries to assess domain-specific PAs, which has the advantage of being able to compare the trend of PA across countries. It can also be done to a large population with less administration and cost in a relatively short time. Lastly, our study has a cross-sectional study design which makes it difficult to examine the causal relationship between domain-specific PA and the risk of diabetes.

## Conclusion

In conclusion, leisure-time PA was inversely associated with prevalence of diabetes in both Korean men and women, while transport PA was associated only in men. Moreover, PA of vigorous intensity, compared to moderate intensity, and inclusion of resistance exercise may confer additional benefits in preventing diabetes in Korean adults. Our findings provide new evidence that domain-specific PA may act differently in the development of diabetes. More studies are needed to confirm our findings in diverse ethnic populations.

## Supplementary Information

Supplementary Information.
