# Supplementary material for: Association between domain-specific physical activity and diabetes in Korean adults
Source: Sci Rep. 2021 Jun 22;11:13066. doi: 10.1038/s41598-021-92560-x (PMC8219728; doi:10.1038/s41598-021-92560-x)
Supplement: Supplementary file 1 — Supplementary Information. [file 41598_2021_92560_MOESM1_ESM.docx]

**Supplementary Materials**

**Association between domain-specific physical activity and diabetes in Korean adults**

Eun-Byeol Lee^1, 2, 3^, Sunghyun Hong^1, 2^, Jihee Min^1, 2^, Dong-Hyuk Park^1, 2^, Wonhee Cho ^1, 2^, Sang-Hoon Suh^3, 4^, Hae-Dong Lee^3, 4^, Han-Joo Lee^3, 4^, Heejin Kimm^3, 5^, Sun Ha Jee^2, 3, 5^, Eun Seok Kang^3, 6^, Dong Hoon Lee^7^*, Justin Y. Jeon^1, 2, 3^*

^1^Department of Sport Industry Studies, Yonsei University, Seoul, Korea, ^2^Exercise Medicine Center for Diabetes and Cancer Patients, ICONS, Yonsei University, Seoul, Korea, ^3^Frontier Research Institute of Convergence Sports Science, Yonsei University, ^4^Department of Physical Education, Yonsei University, Seoul, Korea, ^5^Graduate School of Public Health, Institute for Health Promotion, Yonsei University, Seoul, Korea, ^6^Department of Internal Medicine, Yonsei University College of Medicine, Seoul, Korea, ^7^Department of Nutrition, Harvard T.H. Chan School of Public Health, Boston, MA, USA

**Supplementary Table 1. Odds ratio (95% CI) according to domain-specific physical activity**

|  |  | Model 1 | | Model 2 | | Model 3 | |
| --- | --- | --- | --- | --- | --- | --- | --- |
|  | N | OR | 95% CI | OR | 95% CI | OR | 95% CI |
| **Men** |  |  |  |  |  |  |  |
| **Total PA** |  |  |  |  |  |  |  |
| No | 3523 | ref. |  |  |  |  |  |
| Yes | 7955 | 0.93 | (0.83-1.04) | 0.93 | (0.83-1.04) | 0.93 | (0.83-1.04) |
| **WPA** |  |  |  |  |  |  |  |
| No | 10218 | ref. |  |  |  |  |  |
| Yes | 1259 | 1.08 | (0.90-1.30) | 1.09 | (0.90-1.31) | 1.06 | (0.88-1.28) |
| **LPA** |  |  |  |  |  |  |  |
| No | 7558 | ref. |  |  |  |  |  |
| Yes | 3914 | **0.88** | (0.78-0.99) | **0.88** | (0.78-0.99) | **0.86** | (0.76-0.97) |
| **TPA** |  |  |  |  |  |  |  |
| No | 5477 | ref. |  |  |  |  |  |
| Yes | 5979 | 0.91 | (0.82-1.01) | 0.91 | (0.82-1.01) | 0.92 | (0.83-1.03) |
| **Women** |  |  |  |  |  |  |  |
| **Total PA** |  |  |  |  |  |  |  |
| No | 4776 | ref. |  |  |  |  |  |
| Yes | 10399 | **0.90** | (0.80-1) | 0.91 | (0.82-1.02) | 0.95 | (0.85-1.07) |
| **WPA** |  |  |  |  |  |  |  |
| No | 13930 | ref. |  |  |  |  |  |
| Yes | 1235 | 0.93 | (0.74-1.16) | 0.94 | (0.75-1.18) | 0.90 | (0.72-1.14) |
| **LPA** |  |  |  |  |  |  |  |
| No | 11698 | ref. |  |  |  |  |  |
| Yes | 3473 | **0.71** | (0.61-0.82) | **0.72** | (0.62-0.84) | **0.77** | (0.65-0.89) |
| **TPA** |  |  |  |  |  |  |  |
| No | 6233 | ref. |  |  |  |  |  |
| Yes | 8897 | 0.99 | (0.89-1.10) | 1.00 | (0.90-1.12) | 1.03 | (0.93-1.15) |

Model 1: Adjusted for age, Model 2: Adjusted for Model1 + DM family history, alcohol consumption, smoking, income, education, sedentary time, Model 3: Adjusted for Model 2 + BMI

Abbreviation: ref, reference; PA, physical activity; WPA, work related physical activity; LPA, leisure-time physical activity; TPA, transportation physical activity.

**Supplementary Table 2. Odds ratio (95% CI) according to domain-specific physical activity (analysis restricted to undiagnosed DM)**

|  | Men | Model 1 | Model 2 | Model 3 | Women | Model 1 | Model 2 | Model 3 |
| --- | --- | --- | --- | --- | --- | --- | --- | --- |
|  | n | OR (95% CI) | OR (95% CI) | OR (95% CI) | n | OR (95% CI) | OR (95% CI) | OR (95% CI) |
| Total PA |  |  |  |  |  |  |  |  |
| No | 3523 | ref. |  |  | 571 | ref. |  |  |
| Yes | 7955 | 0.93(0.83-1.05) | 0.93(0.83-1.04) | 0.93(0.83-1.05) | 868 | 0.94(0.84-1.06) | 0.95(0.84-1.07) | 1.00(0.89-1.13) |
| WPA |  |  |  |  |  |  |  |  |
| No | 10218 | ref. |  |  | 1360 | ref. |  |  |
| Yes | 1259 | 1.06(0.87-1.29) | 1.05(0.86-1.28) | 1.02(0.83-1.25) | 76 | 0.91(0.71-1.16) | 0.91(0.71-1.17) | 0.87(0.68-1.12) |
| LPA |  |  |  |  |  |  |  |  |
| No | 7558 | ref. |  |  | 1260 | ref. |  |  |
| Yes | 3914 | 0.89(0.78-1.01) | 0.89(0.78-1.01) | 0.87(0.76-0.99) | 179 | 0.70(0.59-0.83) | 0.71(0.60-0.84) | 0.76(0.64-0.90) |
| TPA |  |  |  |  |  |  |  |  |
| No | 5477 | ref. |  |  | 650 | ref. |  |  |
| Yes | 5979 | 0.90(0.81-1.01) | 0.90(0.80-1.01) | 0.92(0.82-1.02) | 783 | 1.01(0.90-1.13) | 1.02(0.91-1.15) | 1.06(0.94-1.19) |

Model 1: Adjusted for age, Model 2: Adjusted for Model1 + DM family history, alcohol consumption, smoking, income, education, sedentary time, Model 3: Adjusted for Model 2 + BMI, Abbreviation: PA, physical activity; WPA, work related physical activity; LPA, leisure-time physical activity; TPA, transportation physical activity; ref, reference.

**Supplementary Table 3. Odds ratio (95% CI) according to domain-specific physical activity (analysis restricted to undiagnosed DM)**

|  |  | Model 1 | Model 2 | Model 3 |  | Model 1 | Model 2 | Model 3 |
| --- | --- | --- | --- | --- | --- | --- | --- | --- |
|  | Men | OR (95% CI) | OR (95% CI) | OR (95% CI) | Women | OR (95% CI) | OR (95% CI) | OR (95% CI) |
| Total PA (METs-min/wk) | | |  |  | n |  |  |  |
| 0 | 552 | ref. | ref. | ref. | 571 | ref. | ref. | ref. |
| <600 | 336 | 1.00(0.86-1.16) | 1.00(0.86-1.16) | 1.01(0.86-1.18) | 414 | 1.04(0.91-1.20) | 1.05(0.91-1.20) | 1.08(0.94-1.25) |
| ≥600 | 643 | 0.90(0.79-1.02) | 0.89(0.79-1.02) | 0.89(0.78-1.02) | 454 | 0.86(0.75-0.99) | 0.87(0.76-1.00) | 0.93(0.81-1.07) |
| WPA (METs-min/wk) | | |  |  |  |  |  |  |
| 0 | 1398 | ref. | ref. | ref. | 1360 | ref. | ref. | ref. |
| <600 | 46 | 1.14(0.82-1.57) | 1.00(0.86-1.16) | 1.14(0.82-1.59) | 34 | 0.93(0.64-1.34) | 0.94(0.65-1.35) | 0.95(0.66-1.38) |
| ≥600 | 87 | 1.03(0.81-1.30) | 1.01(0.80-1.28) | 0.96(0.76-1.23) | 42 | 0.89(0.64-1.23) | 0.89(0.64-1.23) | 0.82(0.59-1.14) |
| LPA (METs-min/wk) | | |  |  |  |  |  |  |
| 0 | 1128 | ref. | ref. | ref. | 1260 | ref. | ref. | ref. |
| <600 | 166 | 1.04(0.87-1.25) | 1.05(0.87-1.25) | 1.01(0.84-1.22) | 82 | 0.73(0.57-0.92) | 0.73(0.58-0.93) | 0.77(0.60-0.98) |
| ≥600 | 552 | 0.81(0.69-0.94) | 0.80(0.69-0.94) | 0.79(0.67-0.92) | 97 | 0.68(0.55-0.85) | 0.69(0.56-0.86) | 0.76(0.61-0.95) |
| TPA (METs-min/wk) | | |  |  |  |  |  |  |
| 0 | 783 | ref. | ref. | ref. | 650 | ref. | ref. | ref. |
| <600 | 360 | 0.98(0.85-1.12) | 0.97(0.85-1.12) | 1.00(0.87-1.15) | 429 | 1.05(0.92-1.20) | 1.06(0.93-1.21) | 1.10(0.96-1.26) |
| ≥600 | 384 | 0.84(0.74-0.97) | 0.84(0.73-0.96) | 0.85(0.74-0.97) | 354 | 0.97(0.84-1.11) | 0.98(0.85-1.13) | 1.01(0.88-1.17) |

Model 1: Adjusted for age, Model 2: Adjusted for Model1 + DM family history, alcohol consumption, smoking, income, education, sedentary time, Model 3: Adjusted for Model 2 + BMI, bold is p<0.05, Abbreviation: PA, physical activity; WPA, work related physical activity; LPA, leisure-time physical activity; TPA, transportation physical activity; ref, reference.

**Supplementary Table 4. Odds ratio (95% CI) according to total physical activity by subgroups**

| Total PA | Men | | 0 | <600 | ≥600 |  | Women | | 0 | <600 | ≥600 |  |
| --- | --- | --- | --- | --- | --- | --- | --- | --- | --- | --- | --- | --- |
|  |  | |  |  |  |  |  | |  |  |  |  |
|  | Total | DM, *N*(%) | ref. | OR (95% CI) | OR (95% CI) | P  interaction | Total | DM, *N*(%) | ref. | OR (95% CI) | OR (95% CI) | P  interaction |
| Age |  |  |  |  |  | 0.04 |  |  |  |  |  | 0.99 |
| <65 | 8534 | 988 (11.6) | 1 | 0.92 (0.75-1.13) | 0.96 (0.82-1.13) |  | 11372 | 805 (7.1) | 1 | 0.99 (0.81-1.21) | 0.96 (0.80-1.15) |  |
| ≥65 | 2944 | 851 (28.9) | 1 | 1.16 (0.94-1.44) | 0.95 (0.78-1.15) |  | 3803 | 1017 (26.7) | 1 | 1.0 (0.84-1.20) | 0.83 (0.68-1.0) |  |
| BMI |  |  |  |  |  | <0.001 |  |  |  |  |  | 0.33 |
| <23 | 3825 | 483 (12.6) | 1 | 1.23 (0.94-1.60) | 0.96 (0.76-1.21) |  | 7284 | 438 (6) | 1 | 1.33 (1.03-1.71) | 0.93 (0.72-1.21) |  |
| ≥23 | 7527 | 1335 (17.7) | 1 | 0.91 (0.77-1.09) | 0.88 (0.76-1.02) |  | 7724 | 1361 (17.6) | 1 | 0.92 (0.79-1.08) | 0.88 (0.76-1.02) |  |
| Family history of diabetes | | |  |  |  | <0.001 |  |  |  |  |  | <0.001 |
| Yes | 2331 | 612 (26.3) | 1 | 1.10 (0.83-1.46) | 0.89 (0.70-1.12) |  | 3617 | 687 (19) | 1 | 0.87 (0.68-1.10) | 0.83 (0.67-1.04) |  |
| No | 8760 | 1115 (12.7) | 1 | 0.96 (0.80-1.15) | 0.93 (0.80-1.08) |  | 11160 | 1022 (9.2) | 1 | 1.06 (0.90-1.26) | 0.90 (0.76-1.07) |  |
| Income |  |  |  |  |  | 0.63 |  |  |  |  |  | 0.33 |
| ≥Middle | 7961 | 1145 (18.2) | 1 | 1.04 (0.88-1.22) | 0.94 (0.81-1.08) |  | 10931 | 1547 (14.2) | 1 | 1.04 (0.90-1.20) | 0.94 (0.81-1.08) |  |
| Low | 3491 | 391 (11.2) | 1 | 0.99 (0.71-1.38) | 1.01 (0.77-1.32) |  | 4193 | 262 (6.2) | 1 | 0.96 (0.68-1.36) | 0.83 (0.60-1.14) |  |
| Education |  |  |  |  |  | 0.55 |  |  |  |  |  | 0.33 |
| <University | 6916 | 1365 (19.7) | 1 | 1.02 (0.86-1.21) | 0.91 (0.78-1.05) |  | 10203 | 1648 (16.2) | 1 | 1.04 (0.90-1.20) | 0.89 (0.77-1.02) |  |
| ≥University | 4530 | 464 (10.2) | 1 | 1.11 (0.82-1.51) | 1.15 (0.89-1.48) |  | 4931 | 164 (3.3) | 1 | 0.84 (0.53-1.33) | 1.09 (0.73-1.62) |  |
| Smoking |  |  |  |  |  | 0.39 |  |  |  |  |  | 0.12 |
| Ever smoker | 8751 | 1529 (17.5) | 1 | 1.04 (0.88-1.22) | 0.94 (0.82-1.07) |  | 1593 | 157 (9.9) | 1 | 0.93 (0.45-1.17) | 0.76 (0.48-1.19) |  |
| Never | 2658 | 293 (11) | 1 | 0.94 (0.64-1.38) | 1.04 (0.76-1.42) |  | 13475 | 1633 (12.1) | 1 | 1.06 (0.92-1.22) | 0.94 (0.82-1.08) |  |
| Alcohol intake |  |  |  |  |  | 0.04 |  |  |  |  |  | 0.34 |
| < once/1month | 3306 | 620 (18.8) | 1 | 1.01(0.79-1.30) | 0.83(0.67-1.04) |  | 8925 | 1358 (15.2) | 1 | 1.10(0.94-1.28) | 0.93(0.80-1.09) |  |
| ≥once/1month | 8109 | 1203 (14.8) | 1 | 1.02(0.85-1.23) | 1.01(0.87-1.18) |  | 6155 | 440 (7.1) | 1 | 0.83(0.63-1.10) | 0.90(0.70-1.15) |  |
| Sedentary time |  |  |  |  |  | 0.004 |  |  |  |  |  | 0.97 |
| Less | 5253 | 900 (17.1) | 1 | 1.06(0.85-1.31) | 0.93(0.78-1.11) |  | 7161 | 791 (11) | 1 | 0.99(0.81-1.22) | 0.87(0.72-1.05) |  |
| More | 6225 | 939 (15.1) | 1 | 1.00(0.81-1.22) | 0.99(0.83-1.19) |  | 8014 | 1031 (12.9) | 1 | 1.05(0.88-1.26) | 0.98(0.81-1.18) |  |

Data are presented as number(percentage). Multivariable models adjusted for age, DM family history, alcohol consumption, smoking, income, education, sedentary time, BMI. Abbreviation: ref, reference; DM, diabetes mellitus, PA, physical activity.

**Supplementary Table 5.** **Odds ratio (95% CI) according to leisure-time physical activity by subgroups**

| Leisure-time PA | Men | | 0 | <600 | ≥600 |  | Women | | 0 | <600 | ≥600 |  |
| --- | --- | --- | --- | --- | --- | --- | --- | --- | --- | --- | --- | --- |
|  | Total | DM, *N*(%) | ref | OR (95% CI) | OR (95% CI) | P  interaction | Total | DM, *N*(%) | ref | OR (95% CI) | OR (95% CI) | P  interaction |
| Age |  |  |  |  |  | 0.001 |  |  |  |  |  | 0.31 |
| <65 | 8534 | 988 (11.6) | 1 | 1.0(0.80-1.24) | 0.84(0.70-1.02) |  | 11372 | 805 (7.1) | 1 | 0.84(0.64-1.12) | 0.83(0.65-1.07) |  |
| ≥65 | 2944 | 851 (28.9) | 1 | 1.03(0.75-1.43) | 0.86(0.66-1.13) |  | 3803 | 1017 (26.7) | 1 | 0.70(0.47-1.03) | 0.98(0.67-1.42) |  |
| BMI |  |  |  |  |  | 0.12 |  |  |  |  |  | 0.34 |
| <23 | 3825 | 483 (12.6) | 1 | 1.02(0.70-1.49) | 0.94(0.70-1.28) |  | 7284 | 438 (6) | 1 | 0.89(0.58-1.37) | 0.87(0.59-1.29) |  |
| ≥23 | 7527 | 1335 (17.7) | 1 | 1.05(0.86-1.29) | 0.85(0.71-1.01) |  | 7724 | 1361 (17.6) | 1 | 0.75(0.57-0.98) | 0.88(0.69-1.12) |  |
| Family history of diabetes | | |  |  |  |  |  |  |  |  |  |  |
| Yes | 2331 | 612 (26.3) | 1 | 1.06(0.85-1.33) | 0.90(0.75-1.10) |  | 3617 | 687 (19) | 1 | 0.66(0.47-0.91) | 0.81(0.60-1.08) |  |
| No | 8760 | 1115 (12.7) | 1 | 1.06(0.78-1.46) | 0.81(0.62-1.06) |  | 11160 | 1022 (9.2) | 1 | 0.93(0.66-1.31) | 0.86(0.64-1.17) |  |
| Income |  |  |  |  |  | 0.10 |  |  |  |  |  | 0.004 |
| ≥Middle | 7961 | 1145 (18.2) | 1 | 1.08(0.87-1.34) | 0.90(0.75-1.08) |  | 10931 | 1547 (14.2) | 1 | 0.81(0.63-1.05) | 0.98(0.77-1.23) |  |
| Low | 3491 | 391 (11.2) | 1 | 1.01(0.72-1.40) | 0.84(0.64-1.11) |  | 4193 | 262 (6.2) | 1 | 0.66(0.41-1.08) | 0.63(0.42-0.96) |  |
| Education |  |  |  |  |  | 0.13 |  |  |  |  |  | 0.21 |
| <University | 6916 | 1365 (19.7) | 1 | 0.94(0.74-1.19) | 0.85(0.70-1.03) |  | 10203 | 1648 (16.2) | 1 | 0.82(0.64-1.05) | 0.85(0.68-1.08) |  |
| ≥University | 4530 | 464 (10.2) | 1 | 1.32(0.99-1.75) | 0.98(0.77-1.26) |  | 4931 | 164 (3.3) | 1 | 0.65(0.38-1.11) | 0.89(0.57-1.39) |  |
| Smoking |  |  |  |  |  | 0.26 |  |  |  |  |  | 0.36 |
| Ever smoker | 8751 | 1529 (17.5) | 1 | 1.04(0.85-1.26) | 0.87(0.73-1.03) |  | 1593 | 157 (9.9) | 1 | 1.20(0.57-2.52) | 0.49(0.18-1.30) |  |
| Never | 2658 | 293 (11) | 1 | 1.09(0.71-1.67) | 0.95(0.66-1.36) |  | 13475 | 1633 (12.1) | 1 | 0.74(0.58-0.94) | 0.89(0.73-1.10) |  |
| Alcohol intake |  |  |  |  |  | 0.16 |  |  |  |  |  | 0.38 |
| < once/1month | 3306 | 620 (18.8) | 1 | 0.92(0.66-1.29) | 0.76(0.56-1.01) |  | 8925 | 1358 (15.2) | 1 | 0.83(0.64-1.08) | 0.80(0.61-1.03) |  |
| ≥once/1month | 8109 | 1203 (14.8) | 1 | 1.11(0.90-1.37) | 0.93(0.78-1.10) |  | 6155 | 440 (7.1) | 1 | 0.66(0.42-1.03) | 1(0.72-1.39) |  |
| Sedentary time |  |  |  |  |  | 0.09 |  |  |  |  |  | 0.79 |
| Less | 5253 | 900 (17.1) | 1 | 0.96(0.73-1.27) | 0.89(0.72-1.09) |  | 7161 | 791 (11) | 1 | 0.74(0.54-1.03) | 0.82(0.62-1.07) |  |
| More | 6225 | 939 (15.1) | 1 | 1.13(0.89-1.43) | 0.88(0.70-1.09) |  | 8014 | 1031 (12.9) | 1 | 0.80(0.58-1.11) | 0.92(0.68-1.25) |  |

Data are presented as number(percentage). Multivariable models adjusted for age, DM family history, alcohol consumption, smoking, income, education, sedentary time, BMI. Abbreviation: ref, reference; DM, diabetes mellitus, PA, physical activity.

**Supplementary Table 6. Odds ratio (95% CI) according to work physical activity by subgroups**

| Work PA | Men | | 0 | <600 | ≥600 |  | Women | | 0 | <600 | ≥600 |  |
| --- | --- | --- | --- | --- | --- | --- | --- | --- | --- | --- | --- | --- |
|  | Total | DM, *N*(%) | ref. | OR (95% CI) | OR (95% CI) | P  interaction | Total | DM, *N*(%) | ref. | OR (95% CI) | OR (95% CI) | P  interaction |
| Age |  |  |  |  |  | 0.17 |  |  |  |  |  | 0.84 |
| <65 | 8534 | 988 (11.6) | 1 | 1.41(0.97-2.05) | 0.95(0.72-1.25) |  | 11372 | 805 (7.1) | 1 | 0.73(0.44-1.23) | 0.90(0.62-1.31) |  |
| ≥65 | 2944 | 851 (28.9) | 1 | 0.69(0.37-1.30) | 1.23(0.79-1.92) |  | 3803 | 1017 (26.7) | 1 | 1.23(0.76-1.98) | 0.95(0.58-1.57) |  |
| BMI |  |  |  |  |  | 1.00 |  |  |  |  |  | 0.21 |
| <23 | 3825 | 483 (12.6) | 1 | 0.65(0.31-1.36) | 0.78(0.47-1.30) |  | 7284 | 438 (6) | 1 | 0.99(0.51-1.92) | 0.58(0.29-1.17) |  |
| ≥23 | 7527 | 1335 (17.7) | 1 | 1.34(0.94-1.93) | 1.05(0.81-1.36) |  | 7724 | 1361 (17.6) | 1 | 0.91(0.61-1.36) | 1.00(0.72-1.39) |  |
| Family history of diabetes | | |  |  |  | 0.89 |  |  |  |  |  | 0.04 |
| Yes | 2331 | 612 (26.3) | 1 | 1.32(0.81-2.17) | 0.95(0.63-1.44) |  | 3617 | 687 (19) | 1 | 0.79(0.42-1.50) | 0.73(0.43-1.23) |  |
| No | 8760 | 1115 (12.7) | 1 | 0.80(0.50-1.29) | 1.03(0.77-1.37) |  | 11160 | 1022 (9.2) | 1 | 1.02(0.66-1.57) | 0.97(0.66-1.42) |  |
| Income |  |  |  |  |  | 0.96 |  |  |  |  |  | 0.18 |
| ≥Middle | 7961 | 1145 (18.2) | 1 | 1.02(0.70-1.49) | 1.03(0.80-1.33) |  | 10931 | 1547 (14.2) | 1 | 0.92(0.64-1.35) | 0.83(0.60-1.15) |  |
| Low | 3491 | 391 (11.2) | 1 | 1.37(0.74-2.52) | 0.72(0.42-1.24) |  | 4193 | 262 (6.2) | 1 | 0.97(0.41-2.29) | 1.30(0.66-2.59) |  |
| Education |  |  |  |  |  | 0.83 |  |  |  |  |  | 0.52 |
| <University | 6916 | 1365 (19.7) | 1 | 0.99(0.67-1.48) | 0.85(0.64-1.12) |  | 10203 | 1648 (16.2) | 1 | 0.96(0.66-1.40) | 0.87(0.63-1.21) |  |
| ≥University | 4530 | 464 (10.2) | 1 | 1.50(0.87-2.56) | 1.30(0.86-1.97) |  | 4931 | 164 (3.3) | 1 | 0.90(0.36-2.29) | 1.07(0.54-2.15) |  |
| Smoking |  |  |  |  |  | 0.02 |  |  |  |  |  | 0.95 |
| Ever smoker | 8751 | 1529 (17.5) | 1 | 1.07(0.76-1.51) | 0.82(0.63-1.05) |  | 1593 | 157 (9.9) | 1 | 1.37(0.46-4.08) | 0.84(0.36-1.98) |  |
| Never | 2658 | 293 (11) | 1 | 1.42(0.58-3.52) | 2.34(1.40-3.91) |  | 13475 | 1633 (12.1) | 1 | 0.89(0.62-1.28) | 0.89(0.65-1.22) |  |
| Alcohol intake |  |  |  |  |  | 0.28 |  |  |  |  |  | 0.22 |
| < once/1month | 3306 | 620 (18.8) | 1 | 1.23(0.69-2.20) | 0.86(0.56-1.31) |  | 8925 | 1358 (15.2) | 1 | 1.02(0.69-1.49) | 0.77(0.54-1.11) |  |
| ≥once/1month | 8109 | 1203 (14.8) | 1 | 1.06(0.72-1.56) | 1.02(0.78-1.34) |  | 6155 | 440 (7.1) | 1 | 0.60(0.27-1.34) | 1.21(0.73-2.00) |  |
| Sedentary |  |  |  |  |  | 0.16 |  |  |  |  |  | 0.60 |
| Less | 5253 | 900 (17.1) | 1 | 1.26(0.82-1.93) | 0.86(0.65-1.15) |  | 7161 | 791 (11) | 1 | 0.76(0.46-1.23) | 0.88(0.60-1.29) |  |
| More | 6225 | 939 (15.1) | 1 | 0.91(0.56-1.49) | 1.22(0.83-1.79) |  | 8014 | 1031 (12.9) | 1 | 1.15(0.71-1.89) | 0.88(0.56-1.39) |  |

Data are presented as number(percentage). Multivariable models adjusted for age, DM family history, alcohol consumption, smoking, income, education, sedentary time, BMI. Abbreviation: ref, reference; DM, diabetes mellitus, PA, physical activity.

**Supplementary Table 7. Odds ratio (95% CI) according to transport physical activity by subgroups**

| Transportation PA | Men | | 0 | <600 | ≥600 |  | Women | | 0 | <600 | ≥600 |  |
| --- | --- | --- | --- | --- | --- | --- | --- | --- | --- | --- | --- | --- |
|  | Total | DM, *N*(%) | ref. | OR (95% CI) | OR (95% CI) | P  interaction | Total | DM, *N*(%) | ref. | OR (95% CI) | OR (95% CI) | P  interaction |
| Age |  |  |  |  |  | 0.65 |  |  |  |  |  | 0.56 |
| <65 | 8534 | 988 (11.6) | 1 | 1.01(0.84-1.22) | 0.95(0.80-1.13) |  | 11372 | 805 (7.1) | 1 | 1.02(0.84-1.23) | 1.11(0.92-1.33) |  |
| ≥65 | 2944 | 851 (28.9) | 1 | 1.10(0.90-1.34) | 0.83(0.68-1.02) |  | 3803 | 1017 (26.7) | 1 | 1.05(0.88-1.25) | 0.84(0.69-1.02) |  |
| BMI |  |  |  |  |  | 0.37 |  |  |  |  |  | 0.98 |
| <23 | 3825 | 483 (12.6) | 1 | 1(0.77-1.29) | 0.95(0.74-1.21) |  | 7284 | 438 (6) | 1 | 1.40(1.09-1.78) | 0.99(0.75-1.30) |  |
| ≥23 | 7527 | 1335 (17.7) | 1 | 1.04(0.89-1.23) | 0.84(0.72-0.98) |  | 7724 | 1361 (17.6) | 1 | 0.95(0.82-1.10) | 0.99(0.85-1.16) |  |
| Family history of diabetes | | |  |  |  | 0.89 |  |  |  |  |  | 0.05 |
| Yes | 2331 | 612 (26.3) | 1 | 1.03(0.80-1.34) | 0.84(0.66-1.07) |  | 3617 | 687 (19) | 1 | 0.98(0.78-1.23) | 1.08(0.86-1.36) |  |
| No | 8760 | 1115 (12.7) | 1 | 1.04(0.88-1.23) | 0.90(0.76-1.06) |  | 11160 | 1022 (9.2) | 1 | 1.08(0.92-1.28) | 0.97(0.81-1.16) |  |
| Income |  |  |  |  |  | 0.07 |  |  |  |  |  | 0.03 |
| ≥Middle | 7961 | 1145 (18.2) | 1 | 1.02(0.87-1.18) | 0.82(0.71-0.96) |  | 10931 | 1547 (14.2) | 1 | 1.07(0.94-1.24) | 0.99(0.85-1.14) |  |
| Low | 3491 | 391 (11.2) | 1 | 1.09(0.82-1.45) | 1.08(0.82-1.41) |  | 4193 | 262 (6.2) | 1 | 1.01(0.73-1.40) | 1.05(0.76-1.45) |  |
| Education |  |  |  |  |  | 0.85 |  |  |  |  |  | 0.02 |
| <University | 6916 | 1365 (19.7) | 1 | 1.04(0.89-1.22) | 0.85(0.73-1.00) |  | 10203 | 1648 (16.2) | 1 | 1.07(0.93-1.22) | 0.95(0.83-1.10) |  |
| ≥University | 4530 | 464 (10.2) | 1 | 1.01(0.78-1.31) | 0.91(0.71-1.16) |  | 4931 | 164 (3.3) | 1 | 0.94(0.62-1.43) | 1.31(0.89-1.93) |  |
| Smoking |  |  |  |  |  | 0.65 |  |  |  |  |  | 0.17 |
| Ever smoker | 8751 | 1529 (17.5) | 1 | 1.04(0.90-1.21) | 0.85(0.73-0.98) |  | 1593 | 157 (9.9) | 1 | 0.84(0.53-1.32) | 0.93(0.58-1.49) |  |
| Never | 2658 | 293 (11) | 1 | 0.98(0.70-1.38) | 0.98(0.72-1.32) |  | 13475 | 1633 (12.1) | 1 | 1.09(0.95-1.24) | 1.01(0.88-1.17) |  |
| Alcohol intake |  |  |  |  |  | 0.11 |  |  |  |  |  | 0.78 |
| < once/1month | 3306 | 620 (18.8) | 1 | 1.10(0.87-1.38) | 0.77(0.61-0.97) |  | 8925 | 1358 (15.2) | 1 | 1.14(0.98-1.32) | 1(0.85-1.17) |  |
| ≥once/1month | 8109 | 1203 (14.8) | 1 | 0.99(0.84-1.17) | 0.92(0.79-1.08) |  | 6155 | 440 (7.1) | 1 | 0.88(0.68-1.14) | 1.04(0.81-1.34) |  |
| Sedentary time |  |  |  |  |  | 0.18 |  |  |  |  |  | 0.77 |
| Less | 5253 | 900 (17.1) | 1 | 1.10(0.91-1.34) | 0.86(0.72-1.03) |  | 7161 | 791 (11) | 1 | 1.04(0.86-1.27) | 1.00(0.83-1.21) |  |
| More | 6225 | 939 (15.1) | 1 | 0.97(0.81-1.17) | 0.89(0.74-1.08) |  | 8014 | 1031 (12.9) | 1 | 1.08(0.91-1.29) | 1.02(0.84-1.24) |  |

Data are presented as number(percentage). Multivariable models adjusted for age, DM family history, alcohol consumption, smoking, income, education, sedentary time, BMI. Abbreviation: ref, reference; DM, diabetes mellitus, PA, physical activity.

**Supplementary Table 8. Odds ratio (95% CI) according to resistance exercise**

| Resistance Training | |  | Model 1 | | Model 2 | | Model 3 | |
| --- | --- | --- | --- | --- | --- | --- | --- | --- |
|  | n | DM, *N*(%) | OR | 95% CI | OR | 95% CI | OR | 95% CI |
| Men |  |  |  |  |  |  |  |  |
| Not at all | 7739 | 1353 (17.5) | 1 |  | 1 |  | 1 |  |
| 1-day increase | 3719 | 481 (12.9) | **0.96** | (0.93-0.98) | **0.96** | (0.93-0.98) | **0.96** | (0.93-0.99) |
| Women |  |  |  |  |  |  |  |  |
| Not at all | 12705 | 1637 (12.9) | 1 |  | 1 |  | 1 |  |
| 1-day increase | 2452 | 180 (7.3) | **0.93** | (0.89-0.98) | **0.95** | (0.91-1.00) | 0.96 | (0.91-1.00) |

Data are presented as number(percentage). Model 1: Adjusted for age, Model 2: Adjusted for Model 1 + DM family history, alcohol

consumption, smoking, income, education, sedentary time, BMI, Model 3: Adjusted for Model2 + PA time

Abbreviation: DM, diabetes mellitus.
